# A randomized controlled trial to evaluate self-determination theory for exercise adherence and weight control: rationale and intervention description

**DOI:** 10.1186/1471-2458-8-234

**Published:** 2008-07-09

**Authors:** Marlene N Silva, David Markland, Cláudia S Minderico, Paulo N Vieira, Margarida M Castro, Sílvia R Coutinho, Teresa C Santos, Margarida G Matos, Luís B Sardinha, Pedro J Teixeira

**Affiliations:** 1 Department of Exercise and Health, Faculty of Human Kinetics, Technical University of Lisbon, Portugal; 2 School of Sport, Health and Exercise Sciences, University of Wales, Bangor, UK

## Abstract

**Background:**

Research on the motivational model proposed by Self-Determination Theory (SDT) provides theoretically sound insights into reasons why people adopt and maintain exercise and other health behaviors, and allows for a meaningful analysis of the motivational processes involved in behavioral self-regulation. Although obesity is notoriously difficult to reverse and its recidivism is high, adopting and maintaining a physically active lifestyle is arguably the most effective strategy to counteract it in the long-term. The purposes of this study are twofold: i) to describe a 3-year randomized controlled trial (RCT) aimed at testing a novel obesity treatment program based on SDT, and ii) to present the rationale behind SDT's utility in facilitating and explaining health behavior change, especially physical activity/exercise, during obesity treatment.

**Methods:**

Study design, recruitment, inclusion criteria, measurements, and a detailed description of the intervention (general format, goals for the participants, intervention curriculum, and main SDT strategies) are presented. The intervention consists of a 1-year group behavioral program for overweight and moderately obese women, aged 25 to 50 (and pre-menopausal), recruited from the community at large through media advertisement. Participants in the intervention group meet weekly or bi-weekly with a multidisciplinary intervention team (30 2 h sessions in total), and go through a program covering most topics considered critical for successful weight control. These topics and especially their delivery were adapted to comply with SDT and Motivational Interviewing guidelines. Comparison group receive a general health education curriculum. After the program, all subjects are follow-up for a period of 2 years.

**Discussion:**

Results from this RCT will contribute to a better understanding of how motivational characteristics, particularly those related to physical activity/exercise behavioral self-regulation, influence treatment success, while exploring the utility of Self-Determination Theory for promoting health behavior change in the context of obesity.

**Trial Registration:**

**Clinical Trials Gov. Identifier **NCT00513084

## Background

Prevalence of overweight and obesity in modern societies has increased rapidly. Recent reports indicate that 51.6% Portuguese adults are overweight or obese [1]. Obesity is a major public health problem, associated with a number of chronic disease risk factors [2]. Furthermore, overweight and obesity are stigmatizing conditions, especially for women, and are often associated with dysphoric states and psychological problems [3]. Weight loss is recommended as an important part of clinical management and extensive research supports the utility of including physical activity/exercise in weight reduction programs[4]. Unfortunately, the evidence shows that relatively little of the weight loss accomplished in treatment programs is maintained over the long-term [5]. Therefore, there is a strong need for research that identifies predictors of successful weight-loss *maintenance *and tests interventions to specifically promote weight stability after weight loss [6]. Additionally, considering the available evidence regarding behavioral correlates of successful weight loss, comparatively little is know regarding psychological processes associated with long-lasting weight management [7], namely sustained motivation.

The present research project aims at describing a randomized controlled clinical trial (RCT) conceived to test a novel obesity treatment program based on Self-Determination Theory (SDT). Herein, we seek i) to present the theoretical rationale of adopting SDT (and facets of Motivational Interviewing) to facilitate and explain sustained change in physical activity and body weight, and ii) to describe the actual intervention, at a level of detail that effectively allows its evaluation and replication. This is a recommendation from the Behavior Change Consortium [8] and other researchers [9], considering that intervention descriptions are often not specific about the techniques employed and that there is no clear correspondence between theoretical "inspiration" and adoption of particular behavior change techniques. Experimental research and increased theoretical and methodological clarity could accelerate the identification of effective behavior change techniques and the development of evidence-based-practice in health psychology and education. Unfortunately, few examples are currently available in the published peer-reviewed literature.

Primarily, we will evaluate the extent to which a more internal self-regulatory style, higher intrinsic motivation, and more internal perceived locus of causality (regarding exercise behavior) act as mediators of sustained exercise adherence and weight loss. Motives to exercise are also expected to mediate the impact of the intervention on behavioral outcomes; psychological, interpersonal, and health-related motives are hypothesized to positively predict long-term exercise participation. Secondly, individual differences in general causality orientations and perceived treatment climate will be evaluated as moderators of intervention effects; more successful participants are expected to show a more autonomous (less controlling) general orientation style and to describe the treatment interpersonal environment as more autonomy-supportive. A comparison group is included in the research design, to adequately test intervention-specific moderation and mediation hypotheses [10].

## Theoretical rationale

### Self-Determination Theory and motivation for health behavior

Many recent studies have shown the crucial role that motivated behaviors, such as regular exercise, following a healthy diet, and not smoking, can play in the maintenance of health [11]; many people at health risk have the means readily at hand to improve their condition, assuming that they are willing to act. In fact, the failure of many people to adhere to healthy behaviors represents a public health problem, one with many causes but for which a considerable part is motivational in nature. This highlights the need for a clearer understanding of what motivation is and how to facilitate it in the context of health behavior. Motivation refers to the psychological forces or energies that impel a person toward a specific goal. However, for many years, motivation was viewed in a one-dimensional way, as varying only in amount or quantity. Introducing the issue of *quality *of the motivational drive, Deci and Ryan [12,13] developed the Self-Determination Theory (SDT) which distinguishes between *amotivation *(lacking any intention to engage in a behavior), *extrinsic motivation *(where the behavior is engaged in order to achieve outcomes that are separable from the behavior itself) and *intrinsic motivation *(where the behavior is engaged in for the enjoyment and satisfaction inherent in taking part). In addition, SDT distinguishes between qualitatively different forms of extrinsic motivation, by contrasting *autonomous *or *self-determined *vs. *controlled *or *non-self-determined *types of behavioral regulation. This distinction is represented as a continuum and is characterized in terms of the degree to which the regulation of a behavior has been internalized so that it is engaged in with a true sense of volition and choice. Motivation is autonomous to the extent that a person's perceived locus of causality is *internal *(i.e. the perceived source of initiation and regulation for motivated behaviors emanates from the self). Motivation is controlled to the extent that people act because they feel pressured or compelled to do so, either by others or by themselves, and this involves having an *external *perceived locus of causality [14].

**Figure 1:**
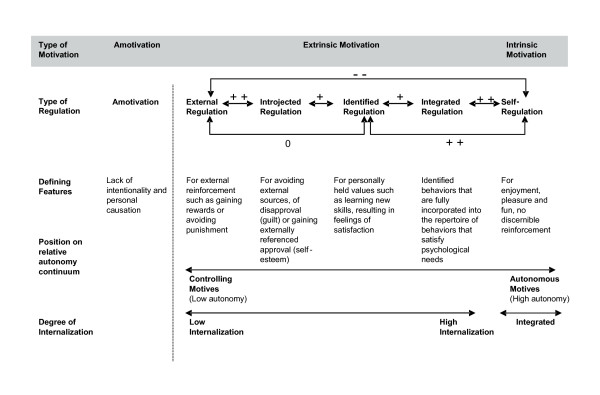
**Model Describing Internalization and Human Motivation**. Model shows different types of motivation and regulation *continuum *reflecting varying degrees of internalization and self-determination. This *continuum *reflects *a simplex-ordered *structure, evident when the correlation between scales measuring adjacent types of motivation such as *external *regulation and *introjected *regulation is higher than the correlation between dimensions that lie further apart such as *external *regulation and *identified *regulation. Adapted from [68].

According to Deci and Ryan, intrinsic motivation is linked to greater productivity, creativity, spontaneity, cognitive flexibility, and perseverance [12]. However, most human behaviors are not intrinsically motivated, which highlights the importance of studying extrinsic forms of motivation. Four types of extrinsic motivation have been described, which can be located on a self-determination continuum [15]. *External regulation *is at the lower end of the continuum. It means doing something in order to gain a reward or to avoid a punishment administered by others. *Introjected regulation *involves an internal feeling of obligation, a need to act in order to avoid feelings of guilt (although the pressure stems from inside, the individual does not feel free regarding the behavior). *Identified Regulation *concerns doing something based on the value of its consequences. In this case, the individual feels free to act and does so because the outcomes are personally important. Finally, when a behavior is coherent with the person's other values, personality schemas and sense of self, the regulation is fully self-determined and said to be *integrated*. SDT proposes that human motivation, vitality, development, and psychological adaptation can be explained by the process of *internalization*, that is, movement along this continuum [13]. A model describing internalization and human motivation is presented in Figure 1.

Autonomous behavior is an expression of one's self and is undertaken with a full sense of choice, accompanied by an internal perceived locus of causality and a sense of true volition. Importantly, according to this theory, autonomously motivated behaviors are more likely to result in meaningful, long-lasting changes. Conversely, behaviors that are controlled by rewards and punishments or by self-imposed pressures are predicted to last only as long as the contingencies or pressures remain in place.

Fortunately, according to SDT, formerly controlled motivation can be internalized and transformed into autonomous motivation, if supportive conditions are in place. In fact, this contemporary theory of human motivation focuses on the psychological nutriments that engender adaptive motivational, behavioral, cognitive, and affective outcomes, by specifying contextual variables that facilitate (or hinder) these processes. The way a person acts in a particular setting cannot be attributed only to individual differences; contextual variables also exert a significant influence. Thus, SDT also considers the influence of the social environment on motivation. According to Deci and Ryan [13], although people have an inherent propensity toward maintaining their well-being, this natural tendency can be thwarted by conditions that frustrate the satisfaction of three basic psychological needs. These needs are for *autonomy *(feeling volitional and feeling choice and responsibility for one's behavior), *competence *(feeling that one can accomplish the behaviors and reach the goal) and *relatedness to others *(feeling understood, cared for and valued by significant others). In the context of health, socio-environmental conditions that facilitate the satisfaction of these needs will promote the internalization of protective and preventive health behaviors so that they are engaged in autonomously and more likely to be maintained in the long term. A recent study in the context of exercise adherence clearly indicated that fulfillment of these three basic needs was related to more self-determined motivational regulations [16]. It can generally be stated that people need to feel a sense of choice and volition with respect to their health-related goals; they need to understand how to attain these goals and feel that they can be effective in carrying out the necessary actions; and feel respected and cared for by practitioners and important others. This means that people not only need to feel that they can carry out a certain behavior (confidence, efficacy, competence), they also need to feel that they are fully responsible for initiating and maintaining that behavior and that they are doing so willingly (autonomy, self-determination, responsibility).

According to SDT, a person will develop and maintain more self-determined motivation when the personal context around them is *autonomy supportive*. The idea of autonomy support refers to eliciting and acknowledging peoples' perspectives, supporting their initiatives, offering choice/options, and providing relevant information, while minimizing pressure and control. Previous research has demonstrated that when physicians are perceived by their patients as being autonomy supportive, patients report greater self-motivation for treatment adherence [17]. Another recent study, designed to test a SDT intervention for motivating tobacco cessation in a clinical trial [18], showed that intervention participants perceived greater autonomy support and reported greater autonomous and competence motivations than did controls, supporting the causal role of autonomy support in the internalization of more internal forms of regulation.

### Self-Determination Theory, exercise and weight control

According to SDT's basic tenets, successful maintenance of weight reduction would occur when people chose eating and exercise behaviors because they personally value weight loss maintenance and its health benefits. Lasting behavior change necessary for maintenance depends not on complying with demands for change but rather on accepting the regulation for change as one's own. In other words, it requires internalizing values and regulation of relevant behaviors and then integrating them with one's sense of self so they become the basis for autonomous regulation. Accordingly, successful weight loss and long-term maintenance would not be achieved if reasons for it were mostly controlling (e.g., because the doctor insisted or based on a strong desire to be thin, according to social norms). Being autonomous in one's relevant actions, that is, having an internal perceived locus of causality, is the crucial predictor of maintained behavior change [19]. In a study specifically designed to study the effects of motivational factors during a weight control program, results showed that the degree of autonomous motivation predicted not only attendance at weekly meetings and weight loss during the program, but, more importantly, maintenance of weight loss at the 23-month follow up [20].

The role of exercise in weight management has also been analyzed in relation to SDT. In a recent study on psychosocial predictors of weight management, increase in intrinsic motivation for physical activity was the strongest predictor of long-term weight change, even after adjusting for initial weight-loss [21]. The authors stressed the importance of ensuring that individuals take on physical activities that they intrinsically enjoy, feel competent at, and that contribute positively to their sense of autonomy as key factors for greater success in the difficult task of long-lasting weight control.

A common theme emerging from the problem of exercise adherence concerns the role of individual's reasons for exercising (participation motives) in determining long-term adherence to regular physical activity. Markland's study findings [22] support the idea that extrinsic motives such as exercising for weight control or because of a doctor's exercise prescription may be perceived as controlling thereby undermining self-determination and leading to a lack of enjoyment of exercise. On the contrary, intrinsic motives, such as exercising for enjoyment may be perceived as informational, enhancing perceptions of self-determination, suggesting that controlling exercise regulations may lead to a greater number of relapses from exercise compared with more self-determined types of exercise regulation. Importantly, exercisers in the maintenance stage of change display significantly more self-determined motivation to exercise than those in the preparation and action stages [23]. Also, others have shown that adherence to exercise in individuals participating in fitness classes is higher when intrinsic motives related to enjoyment and feelings of competence were reported, compared to when body related outcomes (conceptualized as extrinsic) are the primary motivation [24]. Another study on perceived autonomy support for exercise concluded that participants who exercised regularly report modest to large changes in relatedness and competence need satisfaction and intrinsic motivation over time, providing theoretically-sound insights into reasons why people persist with exercise behaviors [25].

The mechanisms that promote self-determined motivation for exercise are now under investigation. Overall enjoyment, perceptions of competence, and intrinsic reasons for participation appear to play a central role in the maintenance of physical activity behaviors [23]. In a large scale RCT, increases in enjoyment mediated physical activity in female adolescents involved in a school-based intervention [26]. In fact, while longitudinal studies are required to better understand the processes underlying maintained exercise behavior, previous results support Markland and Hardy's [27] proposition that an individual's motivational focus needs to shift from extrinsic to intrinsic for long lasting results. New models are needed to understand this shift. We argue that understanding the motivational basis of long-term weight management and exercise requires adopting a more general model of motivation than that offered by the behaviorist tradition, which focuses on controlling a person's behavior with rewards and punishments and on the external determinants of behaviors. It also requires adopting a model more general than that offered by the social cognitive tradition, characterized by a higher emphasis on expected outcomes and enhancing patients' sense of confidence or self-efficacy (quantity of motivation) but not focusing on the quality of motivation, sense of autonomy, and locus of causality [28]. Self-Determination Theory provides such a model.

### The Self-Determination Theory model for maintained behavior change

**Figure 2:**
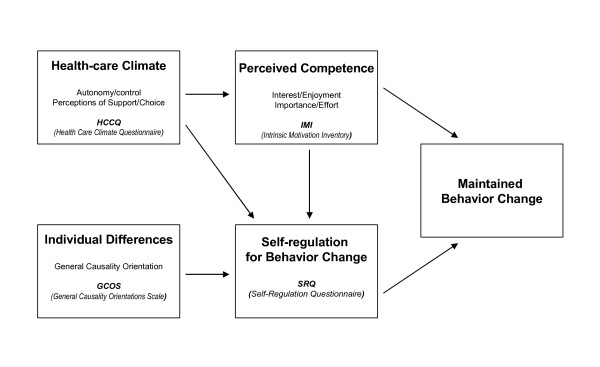
**The Self-Determination Theory Model for maintained behavior change**. Model presents key constructs of health behavior change (scales used also) and the expected relationships among them. Autonomy (both as individual orientation and experimentally promoted) is central to maintained behavior change through is effects on competence and self-regulation. See Methods for abbreviations. Adapted from [29].

Figure 2 presents the self-determination model of health behavior change and the expected relationships among its key constructs. Central to the model is autonomous self-regulation for behavior change. Autonomy support, both experimentally manipulated and as perceived by patients, and also a participant's general autonomy orientation are predicted to enhance autonomous self-regulation and perceived competence. Autonomous self-regulation and perceived competence are in turn expected to increase maintained change of the health-risk behaviors [29].

A recent study [18], aimed at prolonged abstinence from tobacco, was the first to demonstrate that an intervention based on the SDT model facilitates the internalization of motivation, resulting in expected behavioral outcomes. This extends in many ways the previous empirical support for the model found in predicting maintenance of glucose control for diabetes [17], and maintained tobacco abstinence for smokers treated by primary care physicians [30], showing that only when perceived competence is accompanied by the experience of autonomy will it motivate sustained change.

The SDT health care model was formulated to account for the promotion of all health-relevant behaviors, including nutrition and physical activity. Edmunds and colleagues demonstrated that the basic tenets advanced by SDT are amenable to manipulation and revealed the potential utility of SDT to shape behavioral interventions targeting the promotion of healthier and more physically active lifestyles [16]. Given the difficult task of long-lasting weight control, an intervention that could significantly increase the percentage of people able to successfully manage their weight in the long-term, through voluntary, stable changes in diet and physical activity, would represent an important contribution.

We selected SDT as the theoretical basis for this study because it is the only empirically-derived theory of motivation which posits that perceived autonomy (or self-determination) is essential for maintained behavior change and because there are validated psychometric instruments for each construct of the theory. Thus we can test an intervention as well as testing a theory-based process model, assuming the need to treat a theory as a dynamic entity whose form and value rests upon being rigorously applied, tested, and refined [31].

## Methods

### Study design

Randomized controlled trial, analyzing 3-year change in primary outcomes, consisting of a 1-year behavior change intervention and a 2-year follow-up period with no intervention. Participants (n = 259) entered the study in 3 annual cohorts and each cohort was split into 2 randomly-assigned groups: one intervention group and one comparison group. The intervention group attended about 30 group sessions for approximately one year. The comparison group received a general health education curriculum based on several 3 to 6-week long educational topics (e.g. preventive nutrition, stress management, self-care and effective communication skills, among others). The Technical University of Lisbon – Faculty of Human Kinetic Ethics Review Board approved the study.

### Recruitment

#### Inclusion criteria

To be included in the study, participants were required to be female, between 25–50 years old, pre-menopausal, have a BMI between 25 and 40 kg/m^2^, be willing to attend weekly meetings (during 1 year) and be tested regularly (during 3 years); be free from major illnesses, not taking (or having taken in the previous year) medication known to interfere with body weight regulation, namely anti-depressive medication; and willing to not participate in any other formal or informal weight loss program during the first year of the study (intervention group only). Prior to participation, all participants gave written informed consent.

Participants were recruited through a website, newspapers, TV and radio ads, and with flyers distributed in health care centers, local services, schools, etc., asking candidates to enroll in a group-based behavioral (i.e., no medication involved) weight management program. All women who called to inquire about the study were invited to one of several recruitment sessions in which the study was described in more detail, including inclusion/exclusion criteria. Of the 943 women who attended recruitment sessions, 462 were excluded because they failed to comply with the inclusion criteria, primarily related to BMI/age limits; 481 met inclusion criteria but only 290 ultimately committed to the study and were contacted to schedule their baseline measurements; 258 completed initial assessments and entered the study.

### Intervention

#### General format

The intervention group met regularly with the intervention team, in groups of 25–30 participants, for the face-to-face phase of the intervention, for approximately one year (30 meetings in all). These meetings were weekly or bi-monthly, lasted about 120 minutes, and included educational content where physical activity, nutrition, and behavior change specialists presented participants with information, and also conducted interactive discussion, and small group activities. The intervention team comprised 6 Ph.D. or M.S. level exercise physiologists, nutritionists/dietitians, and psychologists. Each session had "check in" and "check out" periods. A comprehensive workbook was produced specifically for this study to provide participants with a written companion manual to complement the face-to-face intervention.

#### Intervention curriculum

##### Physical activity and exercise

Participants were encouraged to find situations in their lives that could be changed in order to increase their caloric expenditure. This could be done in formal (e.g. health clubs) or informal (e.g. daily transportation) settings. Although most activity was home or community-based and unsupervised, some sessions included brief periods of physical activity and participants experimented with a variety of individual and group activity classes. All participants were offered a pedometer to monitor their daily steps. Topics on physical activity included: planning and implementing a structured exercise plan to reach caloric expenditure goals; increasing daily walking and lifestyle physical activity (e.g., using the stairs more often); dealing with safety, weather, and equipment issues; overcoming typical barriers to exercise (time, boredom, lack of facilities); how to monitor exercise intensity, among others. One main goal in this area was to encourage participants to find the activities they enjoyed the most and were more likely to retain for the future. Dance classes and a "physical activity challenge program" were developed to prompt fun, enjoyment, reaching new goals, and experimenting with new activities.

##### Nutrition and eating behavior

The initial emphasis of this program focused on inducing some initial weight loss, which is achieved with a sustained energy deficit, primarily by reducing energy intake. There was also a focus on increasing nutrition knowledge and establishing eating patterns more likely to help subsequent weight maintenance. Specific strategies/goals included: decrease daily caloric intake by 300–400 kcal; improve the overall nutritional quality in the diet; include breakfast and increase the number of meals throughout the day; avoid hunger and uncontrolled intake periods; reduce emotional and "distracted" eating; reduce the amount of food in accordance with energetic demands; prefer low energy density, high-satiety foods; reduce fat in the diet; increase the intake of fruits and vegetables, non-processed cereals and other high fiber foods; reduce the amount of highly processed food and added sugars; consistently read and understand food labels.

#### Cognitive and behavioral aspects

##### Addressing motivation and overcoming barriers

This component focused on identifying and addressing problem areas and difficulties related to the cognitive (attitudinal, motivational) and behavioral changes expected to occur during the program. The motivational aspect of the intervention was deemed as particularly important and was designed to identify personal resistances and barriers and provide skills to prevent, recognize, and overcome expected lapses and relapses, cultivate self-motivation, and encourage self-monitoring. To achieve these goals, a special focus was placed on stressing the importance of understanding internal and external/social determinants of one's behavior and increasing self-awareness towards the most significant individual barriers to the adoption of healthier behaviors. Critical areas were emotional eating, exercise motivation, and formulating adequate goals for weight loss. Specific strategies included increasing self-efficacy (e.g., sharing testimonials from successful individuals under similar circumstances, and setting realistic and attainable goals), analyzing and overcoming typical barriers such as lack of time, lack of knowledge and skills, promoting contingency plans for situations likely to affect compliance, seeking regular social support, dealing positively and constructively with lapses, and building a personal rewards system that consistently acknowledged success and the achievement of individual goals.

##### Increasing knowledge

Before making any changes, participants needed to know why they were necessary and recognize what the purpose of each intervention component was. The goal of this component relied on providing participants with diverse but specific information about issues relevant for weight control and offering women a sound rational for change. This was conducted in a context that would empower participants and support their sense of competence and autonomy regarding health choices and behaviors. Two examples are the principles of energy balance, that is, how body fat stores are regulated based on caloric intake and expenditure, and the limits and pitfalls of popular and restrictive diets.

##### Improving body image

Interventions to improve body image may be helpful adjuncts for people undergoing weight loss, whose concerns extend beyond strictly physical health [32]. Obese persons who start weight reduction programs in pursuit of the ideal physique must confront and come to terms with real limits in their biological and behavioral capacities to meet their goals. Establishing more realistic goals for themselves and their weight/body is an important issue. The main goal in this area was to help participants understand the concept of body image and recognize social and personal threats to their own body image development, stressing that body image is a subjective, psychological construct and that physical appearance and body image can be independent. Persons with negative body image often attribute their life difficulties to their appearance; recovery may be facilitated if participants abandon the idea that they must look different to be happier, attributing negative reactions from others to prejudice rather than to defects in personal traits. Secondly, participants were asked to keep a self-monitoring diary identifying current examples of negative self-statements about physical appearance and negative "body talk", as well as their emotional and behavioral consequences. Third, we worked on cognitive restructuring, helping participants to identify maladaptive assumptions about their appearance, promoting the evaluation of evidence for and against their beliefs and the construction of alternative thoughts. In order to achieve these objectives, relaxation and dance classes were provided to all participants, prompting a more self-conscientious and positive relationship between mind and body.

#### Promoting self-determination

The intervention was designed to establish an autonomy-supportive climate for the participants, regardless of their stage of change or their expectancies and efficacy. The overall goal was to bring each participant closer to making autonomous decisions about whether she wanted to change and how, and then help her cope with the consequences of her choices, whether she succeeded or failed. The following strategies were used as facilitators in enhancing autonomous motivation [15]:

**a) Offering a clear rationale **to adopt a specific behavior; presenting clear contingencies between behavior and outcome; building sustainable knowledge that supported informed choices, by using neutral language during interpersonal communication (e.g., "may" and "could", and not "should" or "must"); and acknowledging participants' feelings and perspectives.

**b) Acknowledging internal conflict **(usual patterns and habits vs. desire to adopt a new behavior); promoting opportunities for participants to indicate their reasons to change activity and nutrition patterns; exploring **perceived benefits and personal barriers**. Such decisional balance constructs have been shown to be important mediators of motivational readiness [33,34].

**c) Providing participants with a menu of options **and a variety of avenues for behavior change, supporting the presentation of tasks and choices with a clear rationale. Because different people have different behavioral preferences and barriers, this is expected to lead to greater long-term adherence, allowing each participant higher congruence between their values and goals, and their lifestyles.

**d) Promoting competence **by practicing skills necessary for completion of specific tasks, such as exercising at a given intensity or reading food labels. More than merely increasing knowledge, the aim was to promote the adoption of self-management skills such as self-monitoring, goal setting, building contingency plans, and managing time (reallocate time to spend it in ways that are more consistent with personal priorities). The intervention team consistently focused on participants' strengths, affirmed small steps as they were taken, and reinforced positive change, knowing that the feeling of competence grows from feedback inherent to the task (cues for objective success), social feedback (comments from others or comparisons to standards), and progression toward a distal goal.

**e) Avoiding the use of external incentives **and controlling, non-informational forms of feedback. Rewards, threats, external evaluation, and deadlines have been shown to undermine intrinsic motivation [35]; rewards and feedback that do not support competence and promote an external perceived locus of causality will undermine autonomy and self-regulation.

**f) Give positive feedback**. Research has shown that feedback as a verbal reward usually enhances intrinsic motivation because it affirms personal competence [15]. This is in accordance with Cognitive Evaluation Theory (a sub-theory of SDT about how social contexts affect motivation), which hypothesizes that perceived competence enhances intrinsic motivation in the context of self-determination. This can be achieved by simply informing people about their performance and avoiding the use of pressuring language, by instructing people how to self-administer informational feedback, and by structuring feedback in a way that does not imply evaluation.

**Figure 3:**
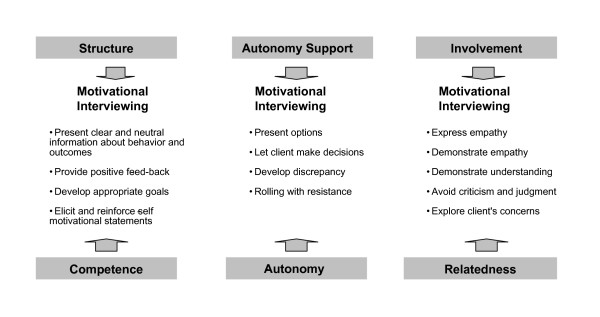
**Relations Between Psychological Needs and Motivational Styles From Self-Determination Theory and the Core Principles from Motivational Interviewing**. Parallels between MI main strategies and factors that are considered in SDT to facilitate integration are striking, providing a theoretical framework for understanding how change occurs. The construct of need satisfaction, as conceptualized within SDT, provides a useful way to understand the positive effects of motivational interviewing. Adapted from [38].

Many of the previous strategies are concordant with **Motivational Interviewing **(MI) [36], a counseling method aimed at promoting behavior change which has successfully been applied to a wide range of health behaviors [37]. Markland, Ryan, Tobin and Rollnick [38] and Vansteenkiste and Sheldon [39] have detailed the parallels between MI and SDT, showing that SDT's theoretical focus on the internalization of therapeutic change and on need satisfaction is fully compatible with key principles and clinical strategies within MI. MI involves avoiding controlling behaviors such as argumentation and direct persuasion for change. Instead, the approach seeks to empower participants to pursue change by eliciting their own personal reasons for change, expressing empathy, supporting self-efficacy, "rolling with" resistance, and helping them to become more aware of discrepancies between goals and actions. According to Markland et al[38] and Vansteenkiste & Sheldon [39] the construct of *need satisfaction*, as conceptualized within SDT, provides a useful way to understand the positive effects of motivational interviewing. MI's key components [33] may be interpreted in terms of the satisfaction of SDT's three needs by its provision of support for autonomously motivated change, presentation of a clear structure to the behavior change environment, and an engaged and involved relationship with the client so that he or she feels personally understood and accepted (see Figure 3). Thus, MI's practical techniques can help to translate SDT's concept of the facilitation of the process of internalization by need satisfaction into specific therapeutic practices and present new ways of testing and developing SDT.

### Measurements

In this trial, assessments were planned to occur at baseline, 4, 12, 16, 24, and 36 months. Besides physical activity, diet, and physiological and psychosocial variables (all described below), demographic variables including menopausal status, age, education level, job or occupation, and socio-economic status were assessed via self-report.

**Weight **was measured twice, to the nearest 0.1 kg (average was used) using an electronic scale (SECA, model 770, Hamburg, Germany) and height is also measured twice, to the nearest 0.1 cm (average was used). Body mass index (BMI) in kilograms per squared meter was calculated from weight (kg) and height (m). **Body composition **was assessed by dual energy x-ray absorptiometry, DXA (QDR-1500; Hologic, Waltham, MA, USA, software version 5.67). Assessment of anthropometric measurements also included body circumferences, total body, trunk, and abdominal fat mass, total body and appendicular fat-free mass. **Resting metabolic rate **was measured using the MedGem portable device (MedGem^®^, Health TechTM Inc., Golden, CO, USA).

**Physical activity and exercise **was measured objectively with the GT1 accelerometer (Actigraph LLC, Pensacola, FL, USA), which quantifies activity counts for every day of the week and estimates free-living PA and energy expenditure at different intensity levels. Participants also concurrently carried a Yamax Digi-Walker SW-200 pedometer (New Lifetyles, Lee's Summit, MO, USA) for assessment of daily steps. This pedometer is a highly accurate and a reliable step counter and is effective for both walking and running [40,41]. In addition, the 7-day Physical Activity Recall (7-d PAR) was used, in interview format, asking subjects to list all activities of moderate, hard, and very hard intensity, as well as daily walking minutes [42,43]. For this calculation, the time spent in each category (hours) is multiplied by body weight and a caloric, or MET value (based on intensity) to yield total exercise energy expenditure.

**Dietary intake **was assessed with three 24-h diet records (randomly assigned days each testing period, including one weekend and two week days). Diet records are analyzed for energy and nutrient intake using Food Processor SQL (Nutrition Analysis Software Version 7.4, ESHA Research, Salem, OR, USA).

Participants completed a comprehensive battery of **psychosocial measures **covering several areas considered as relevant to weight management [44]. This was conducted in standardized conditions of comfort and silence, with a study technician attending every assessment period. Psychosocial areas included previous diets and weight history variables taken from a *diet/weight history questionnaire *developed specifically for this study. Weight outcome evaluations were assessed by 4 questions derived from the *Goals and Relative Weights Questionnaire (GRWQ) *[45], *body size dissatisfaction *was assessed by the difference between self and ideal body figures selected from a list of 9 female silhouettes of increasing size [46]. Body image was further evaluated with the *Body Shape Questionnaire (BSQ) *[47] and the *Physical Self-Perception Profile (PSPP*) [48]. Physical activity determinants were assessed with the *Exercise Perceived Barriers (EPB)*[49], and *Exercise Self-efficacy (ESE) *[50] scales. Dietary restraint, disinhibition and hunger were assessed with the *Three-Factor Eating Inventory *[51]. Psychological states and traits were also considered. Depression status was assessed with the *Beck Depression Inventory II (BDI-II) *[52] and Anxiety was evaluated with the *State-Trait Anxiety Inventory (STAI) *[53]. Self-esteem was assessed with the *Self-Esteem/Self-Concept Questionnaire *[54]. Quality of Life related to weight was measured with the *Impact of Weight on Quality of Life Questionnaire (IWQOL) *developed by Kolotkin et al. [55]. Health-related quality of life was assessed with the *Medical Outcome Survey Short-Form (SF-36*) developed by Ware [56]. Social support for exercise was measured with the *Exercise Social Support Scale (ESS*) [57]. The following psychosocial questionnaires related to SDT were also included, in order to test the intervention model (see Figure 1).

### Self-Determination Theory related instruments

General causality orientations were assessed with the *General Causality Orientations Scale (GCOS) *[58]. Exercise intrinsic motivation was measured with the *Intrinsic Motivation Inventory (IMI) *[27], adapted to specifically measure the dimensions of exercise interest/enjoyment, perceived competence, effort/importance and pressure/tension.

The *Locus of Causality for Exercise Scale (LCE) *[59] assessed locus of causality for exercise. The *Health Care Climate Questionnaire (HCCQ*) [20] measured participants' perceptions of the degree of autonomy support (vs. controllingness) of the relevant health-care providers. The *Exercise Self-Regulation Questionnaire (SRQ-E) *[60] assessed domain-specific individual differences in types of motivation or regulation (external, introjected, identified, and integrated). Finally, the *Exercise Motives Inventory (EMI-2) *[61,62] assessed exercise participation motives on five dimensions (psychological, interpersonal, health-related, body-related and fitness-related motives), including 14 subscales.

At baseline and treatment's end (12 months) individual interviews of approximately 45 minutes were also conducted. The addition of qualitative information to quantitative data (i.e., psychometric instruments) is an important and innovative aspect of this study's assessment plan. Due to space limitations, interviews are not described in detail herein.

### Statistical analyses

Analysis will be conducted for completers-only and using an intent-to-treat model. Baseline data imputation for missing scores at follow-up will be employed by default. Despite limitations of any imputation strategy, this method is conservative and has been recommended before [63]. The use of these models is very important in clinical trials where completion is less than perfect, since completers-only analyses can be severely biased and underpowered [44].

For analysis of moderators and mediators, we will follow recommendations by Kraemer et al. [10]. Generally, for each primary outcome, linear models with be conducted to test for 1) main effect of intervention (I), 2) main effects of each moderator or mediator (M), 3) interactive effects of M and I, 4) overall effect of intervention (plus high vs. low M) and 5) effect of intervention for sub-groups of subjects at specific levels of M. A moderator will be defined as a baseline variable (thus uncorrelated with intervention) that interacts with intervention assignment to produce significant effects. The overall effect of intervention ("additive" effect of intervention and M) will represent the actual (clinical) impact of intervention participation in the presence of M. Finally, profiles of subjects with particular values of M may be identified who are responsive to this particular SDT-based intervention.

Measures of central tendency and distribution will be examined for body weight and psychosocial variables at the different measurement periods. Repeated measures ANOVA and ANCOVA General Linear Model will be used to assess change in dependent variables and to test for within-and between-group differences. Multiple regression analyses and recursive partitioning (signal detection methodology) [64] will be employed to identify characteristics/profiles associated with success, following the principles enunciated above.

### Sample size and power

Sample size calculations were estimated for primary outcomes for a power of 0.8, and a two-tailed p < 0.05. At 1 year, for physical activity, we assume an increase of 300 ± 500 METs-min/sem (intervention, mean ± SD) and 50 ± 400 METs-min/sem for comparison's. A difference effect size (ES) of 0.56 is obtained, for which a sample of 52 participants/group is needed. For changes in body weight (% change of initial) and relative body fat (% change from initial) estimated ES are large (>0.83) and would require considerably less participants/group. Difference ES for some psychosocial variables were also calculated, with estimates (means and SD) based on our previous work [65], and range from 0.67 to 0.83. At maximum, 36 participants/group would be needed to detect significant differences in these variables between intervention and controls. Assuming a dropout rate of 20%, 120 participants/group will remain at 1-year (our primary endpoint), enough to detect even small differences in PA. The lowest ES observed (for PA, ~0.5) would require ~60 participants/group. In the behavioral sciences, "moderate" and "strong" ES for associations between variables (R^2^x100 or percent variance accounted) have been set at 5% and 10% respectively, which is equivalent to correlations of 0.22 and 0.31 [66]. Thus, our sample size will be adequate even for the lowest (i.e., "moderate") association levels.

## Discussion

Despite the cumulative evidence for the positive role of regular physical activity and exercise in long-term weight management, it remains unclear why only about 20% of individuals seeking weight loss are able to successfully integrate activity behaviors into their lifestyles and achieve long-lasting weight control [6]. Since few data are available concerning exercise motivation in the context of weight control, studies in this domain are critical and we believe further application of SDT in the behavioral treatment of obesity is a potentially fruitful area to explore. On the other hand, although empirical data generally support the efficacy of research using motivational techniques, the underlying mechanisms for change remain largely unexplored. According to Bauman [67], if an intervention is not well implemented, it may not affect the proposed mediators and, as a consequence, outcomes may not be sufficiently improved. According to previous research [18,20,30], SDT should be useful in explaining the dynamics of motivation during the course of obesity treatment, providing direct empirical support for autonomy, competence, and perceived autonomy support as three of the psychological processes through which intensive behavioral treatment might operate.

Key innovative features of this study are: a theory-based intervention based on the principles of Self-Determination Theory, a unique setting and population (the first long-term RCT for weight loss conducted in Portugal), *a priori *selection of putative moderators and mediators responsible for therapeutic change, high emphasis on physical activity/exercise, both as exposure and outcome, a systematic evaluation of a large range of potential determinants of outcomes, objective measurements of physical activity (via accelerometry), and precise measure of body composition changes (via DXA).

Results from this study are expected to contribute to a better understanding of how motivational characteristics, particularly those related to physical activity/exercise, influence treatment success during obesity treatment, while exploring the utility of Self-Determination Theory for promoting health behavior change in this most important context. That theory should evolve based on rigorous empirical evidence and that applied intervention research is one of the best ways to evaluate and refine theory [31].

## Competing interests

The authors declare that they have no competing interests.

## Authors' contributions

MNS and PJT conceived this study and drafted the manuscript. MNS led the design and implementation of the behavioral component of the intervention. LBS and PJT are principal investigators and defined the RCT's design, assessments, and analyses. CSM, PNV, MC, TCS, SSC actively participated in the intervention and in data collection. DM and MM provided critical review of the manuscript. All authors read and approved the final manuscript.

## Pre-publication history

The pre-publication history for this paper can be accessed here:


